# The pitfalls and promise of liquid biopsies for diagnosing and treating solid tumors in children: a review

**DOI:** 10.1007/s00431-019-03545-y

**Published:** 2020-01-03

**Authors:** Ruben Van Paemel, Roos Vlug, Katleen De Preter, Nadine Van Roy, Frank Speleman, Leen Willems, Tim Lammens, Geneviève Laureys, Gudrun Schleiermacher, Godelieve A. M. Tytgat, Kathy Astrahantseff, Hedwig Deubzer, Bram De Wilde

**Affiliations:** 1 Center for Medical Genetics, Ghent University Hospital, Ghent, Belgium; 2 Cancer Research Institute Ghent (CRIG), Ghent, Belgium; 3 Department of Pediatric Hematology, Oncology and Stem Cell Transplantation, Ghent University Hospital, C. Heymanslaan 10, 9000 Ghent, Belgium; 4 INSERM U830, Laboratoire de Genetique et Biologie des Cancers, Research Center, PSL Research University, Institut Curie, Paris, France; 5 SiRIC RTOP «Recherche Translationelle en Oncologie Pediatrique », Translational Research Department, Research Center, PSL Research University, Institut Curie, Paris, France; 6 Department of Pediatric Oncology, Hospital Group, Institut Curie, Paris, France; 7 Princess Máxima Center for Pediatric Oncology, Utrecht, The Netherlands; 8 Department of Pediatric Hematology and Oncology, Charité - Universitätsmedizin Berlin, Berlin, Germany; 9 Berlin Institute of Health (BIH), Berlin, Germany; 10 Neuroblastoma Research Group, Experimental and Clinical Research Center (ECRC), Berlin, Germany

**Keywords:** Liquid biopsies, Pediatric solid tumors, Cell-free DNA profiling

## Abstract

Cell-free DNA profiling using patient blood is emerging as a non-invasive complementary technique for cancer genomic characterization. Since these liquid biopsies will soon be integrated into clinical trial protocols for pediatric cancer treatment, clinicians should be informed about potential applications and advantages but also weaknesses and potential pitfalls. Small retrospective studies comparing genetic alterations detected in liquid biopsies with tumor biopsies for pediatric solid tumor types are encouraging. Molecular detection of tumor markers in cell-free DNA could be used for earlier therapy response monitoring and residual disease detection as well as enabling detection of pathognomonic and therapeutically relevant genomic alterations.

*Conclusion*: Existing analyses of liquid biopsies from children with solid tumors increasingly suggest a potential relevance for molecular diagnostics, prognostic assessment, and therapeutic decision-making. Gaps remain in the types of tumors studied and value of detection methods applied. Here we review the current stand of liquid biopsy studies for pediatric solid tumors with a dedicated focus on cell-free DNA analysis. There is legitimate hope that integrating fully validated liquid biopsy–based innovations into the standard of care will advance patient monitoring and personalized treatment of children battling solid cancers.

**Table:** 

**What is Known:** • * Liquid biopsies are finding their way into routine oncological screening, diagnosis, and disease monitoring in adult cancer types fast.* • *The most widely adopted source for liquid biopsies is blood although other easily accessible body fluids, such as saliva, pleural effusions, urine, or cerebrospinal fluid (CSF) can also serve as sources for liquid biopsies*	
**What is New:** • *Retrospective proof-of-concept studies in small cohorts illustrate that liquid biopsies in pediatric solid tumors yield tremendous potential to be used in diagnostics, for therapy response monitoring and in residual disease detection.* • *Liquid biopsy diagnostics could tackle some long-standing issues in the pediatric oncology field; they can enable accurate genetic diagnostics in previously unbiopsied tumor types like renal tumors or brain stem tumors leading to better treatment strategies*

**What is Known:**

• * Liquid biopsies are finding their way into routine oncological screening, diagnosis, and disease monitoring in adult cancer types fast.*

• *The most widely adopted source for liquid biopsies is blood although other easily accessible body fluids, such as saliva, pleural effusions, urine, or cerebrospinal fluid (CSF) can also serve as sources for liquid biopsies*

**What is New:**

• *Retrospective proof-of-concept studies in small cohorts illustrate that liquid biopsies in pediatric solid tumors yield tremendous potential to be used in diagnostics, for therapy response monitoring and in residual disease detection.*

• *Liquid biopsy diagnostics could tackle some long-standing issues in the pediatric oncology field; they can enable accurate genetic diagnostics in previously unbiopsied tumor types like renal tumors or brain stem tumors leading to better treatment strategies*

## Introduction

The analysis of circulating cell-free nucleic acids is being introduced in several medical fields. In obstetrics, non-invasive prenatal aneuploidy screening for trisomy 21 is well established and widely implemented with high sensitivity and specificity [1]. In transplantation medicine, the amount of circulating donor-derived cell-free DNA in the recipient is being explored as a surrogate marker for cellular damage in the donated organ [23, 26]. The analysis of tumor-derived cell-free DNA and RNA is emerging as an alternative to or complementary assay for molecular genetic analyses in tumor tissue biopsies. Commonly referred to as liquid biopsies, the most widely adopted source is blood although other easily accessible body fluids, such as saliva, pleural effusions, urine, or cerebrospinal fluid (CSF), can also serve as sources for liquid biopsies [27]. Moss et al. used cell type-specific methylation to track cell origin, identifying 55% of cell-free DNA in healthy individuals as originating from white blood cells, with contributions from erythrocyte progenitors (30%), vascular endothelial cells (10%), and hepatocytes (1%) [47]. Cell-free nucleic acids are thought to originate from apoptotic or necrotic tissue under physiological and pathological circumstances, but exact biological origins and roles are still under investigation (reviewed in [66]). The fraction of cell-free DNA originating from the tumor is sometimes referred to as circulating or cell-free tumor DNA both abbreviated as ctDNA, which we will use throughout this review. Circulating tumor cells and extracellular vesicles (30–100 nm diameter) originating from tumor cells known as exosomes are other biological sources for DNA, RNA, and proteins in liquid biopsies. This review is limited to the analysis of ctDNA, the most widely adopted fraction. Siravegna [61] and Wan [73] have comprehensively reviewed how other types of liquid biopsies can be exploited to guide patient care, while Merker et al. [61] reviewed the current information about clinical ctDNA assays. We refer the reader to these reviews for more information on those topics.

## Opportunities for liquid biopsies in pediatric oncology

**Fig. 1:**
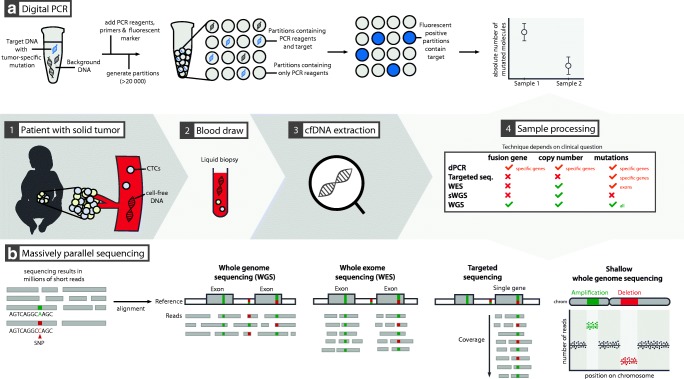
The optimal technique for cell-free DNA (cfDNA) analysis is chosen depending on the clinical question at hand. Commonly used techniques are digital PCR (dPCR) and massively parallel, or “next-generation,” sequencing. Digital PCR (panel **a**), with its unmatched sensitivity, is suited for monitoring known (hotspot) mutations, can be used to detect amplifications or losses of one or two pre-specified genomic regions and to detect pre-defined sites of genomic fusion. Massive parallel sequencing (panel **b**) is useful to detect all types of genomic alterations, depending on the sequencing strategy used. It can evaluate single nucleotide variants (SNVs), copy number aberrations (CNA), genomic fusions or a combination thereof. Whole-genome sequencing (WGS) results in uniform coverage across the entire genome. When performed at low coverage, the technique is termed shallow WGS, and is a cost-effective method to detect CNAs. Performed at higher coverage, the detailed analysis of mutations or translocations on a genome-wide scale is feasible. Whole-exome sequencing (WES) focuses the sequencing effort on the coding regions of the genome, but non-coding or structural variation is largely missed. Targeted sequencing will result in extremely high coverage over a small proportion of the genome, allowing the detection of variants in that specific region with high sensitivity. PCR, polymerase chain reaction. CTC, circulating tumor cells

The discovery of ctDNA dates back to 1977 [40]. However, technological advances have only recently made routine and sensitive analysis feasible, with the advent of the digital polymerase chain reaction (PCR, Fig. 1a) and massively parallel sequencing (Fig. 1b) [27, 61]. The liquid biopsy approach has significant theoretical advantages over classical biopsies, which are often invasive, costly, and potentially harmful to patients. Liquid biopsies are relatively simple to obtain, making them less invasive and less expensive. The patient also benefits from the potential for improved care through finer time-resolved diagnostic monitoring. Liquid biopsies can facilitate increased diagnostic accuracy and enable therapy response monitoring and minimal residual disease (MRD) detection for solid tumors. Tissue biopsy also only reflects a subpopulation of the tumor cells, creating sampling bias. Liquid biopsy better detects spatial or subclonal tumor heterogeneity [15, 25]. Liquid biopsies also enable monitoring of tumor clonal evolution and detection of therapy-relevant novel mutations arising during treatment. Early cancer detection through liquid biopsy is also emerging as a way to perform population surveillance [2]. For many pediatric tumor types, it remains unknown if and to what degree liquid biopsy–based tests will contribute to diagnosis, treatment stratification, and follow-up monitoring. We will discuss the current state of liquid biopsy applications in pediatric oncology and indicate where opportunities can be found.

## Screening and early diagnosis

Little is known about the role of liquid biopsies in cancer screening for the pediatric population.

Screening for cancer requires an easy, low-cost, and low-impact test with a minimalized false detection rate. A test with very high sensitivity and specificity will still detect many false positives if a disease it screens for is very rare, as is the case for cancer in children. As an example, a hypothetical screening test with 99.9% specificity and sensitivity for neuroblastoma, which affects 9–12 children (< 15 years) per million, would generate 988 false positives per 1000 positively screened cases when screening all children under 15 years of age in the general population. Earlier attempts to screen populations for neuroblastoma by ultrasound [51, 57, 76] did not reduce mortality, but overdiagnosed benign adrenal masses as neuroblastomas. The utility of cancer screening in the general pediatric population remains dubious, since cancer screening only becomes clinically relevant if patient benefit can be achieved. Either preventive therapeutic options taken to avoid cancer development (e.g., colectomy in patients with familial adenomatous polyposis) or improved outcome due to treatment after early tumor detection can make screening useful. It is currently unclear what the gold standard is for early cancer detection programs in children with cancer predisposition syndromes stemming from gene mutations in the germline, such as Li-Fraumeni (*TP53*), Von Hippel Lindau (*VHL*), familial adenomatous polyposis (*APC*), DICER1 syndrome or subtypes of primary immunodeficiencies [34]. Liquid biopsies may play a role in this setting in the (near) future, but much controversy remains about correct follow-up since studies with high-quality follow-up remain scarce [39, 54, 65, 75]. Liquid biopsy–based screening could potentially be more accurate, simpler, and associated with less discomfort than current monitoring protocols. Children affected by constitutional mismatch repair deficiency, whose chance of developing cancer by 18 years of age is estimated to be as high as 80 percent [75], could benefit from early detection.

## Diagnostics and therapeutic stratification

Data describing the diagnostic utility of liquid biopsies in pediatric oncology is accumulating. One multi-entity study by Kurihara et al. detected cell-free DNA in plasma samples from all 44 patients with a diverse range of pediatric tumors, but could not definitively show it was tumor-derived because of the paucity in genomic alterations [38]. No large-scale multi-entity studies assessing the diagnostic potential of ctDNA in patients with pediatric solid tumors have been completed to date. However, ultralow passage whole-genome sequencing conducted on 45 pediatric diagnostic pretreatment plasma samples [36] demonstrated the presence of ctDNA in more than half of samples from patients with osteosarcoma, neuroblastoma, Wilms tumor, and alveolar rhabdomyosarcoma. Changes in cell-free DNA plasma load also correlated with treatment response, with higher loads detected in patients with progressive disease. We discuss the studies conducted for single cancers in the context of each disease below. Further evidence of the feasibility of ctDNA detection across different pediatric cancers and different biological sources will come from the ongoing NGSkids (NCT02546453) and MICCHADO (NCT03496402) trials.

### Neuroblastoma

Recent evidence demonstrates that copy number alterations, a mandatory analysis for risk stratification, can be determined from cell-free DNA in blood plasma from neuroblastoma patients [14, 68]. Chromosomal copy number profiles assessed from ctDNA by shallow whole-genome sequencing (at 0.4-fold genomic coverage) were highly concordant with profiles generated from the gold standard, array-based comparative genomic hybridization from the primary tumor biopsy. Work by Combaret et al*.* showed that the two activating *ALK* mutations commonly occurring in neuroblastomas can be detected in plasma by droplet digital PCR with high sensitivity and specificity (90–100%), producing results concordant to those achieved with deep sequencing [18]. Quantitative PCR-based detection of *MYCN* amplifications in peripheral blood from neuroblastoma patients was proven feasible in 2002, before the concept of cancer liquid biopsies was established [17]. Detection sensitivity and specificity is further improved by droplet digital PCR [41]. In all four of the above-mentioned studies, genomic alterations were detected in circulating cell-free DNA that were not detectable in the primary tumor biopsy, suggesting that liquid biopsy diagnostics may be better at capturing tumor heterogeneity or detecting alterations present in metastases.

### Ewing sarcoma

The diagnostic hallmark for Ewing sarcoma is a rearrangement involving the *EWSR1* gene, most commonly *EWSR1*-*FLI1* and *EWSR1*-*ERG* rearrangements, while other rare translocation partners have been reported. *EWSR1* fusion genes can be detected in circulating cell-free DNA with droplet digital PCR or targeted sequencing, providing a liquid biopsy–based diagnostic strategy [37, 60].

### Lymphomas

Although no detailed genomic analysis was conducted, two studies detected significantly higher cell-free DNA loads in plasma from 201 pediatric patients with various lymphoma subtypes [49] and 155 patients with Hodgkin lymphoma [55] as compared with plasma from healthy controls. High circulating cell-free DNA levels correlated with poor prognosis in patients with Hodgkin lymphoma [49], and are present at diagnosis in plasma from patients with B cell non-Hodgkin lymphoma, but decrease during treatment [43]. Pathognomonic *NPM-ALK* fusion genes are readily detectable in plasma from patients with anaplastic large cell lymphoma [49].

### Renal tumors

Pediatric renal tumors are most often not biopsied due to the risk of tumor rupture, which would spill tumor cells into the peritoneal cavity and require treatment intensification. This lack of histological confirmation at diagnosis can lead to misdiagnosis and suboptimal treatment of non-Wilms type tumors. Jimenez et al. [33] retrospectively examined plasma samples collected at diagnosis of different renal tumor types in 18 patients. Tumor-specific copy number and/or single-nucleotide alterations were detected in plasma from all but one patient. Molecular characterization of kidney tumors from plasma samples collected at diagnosis could, therefore, open the door to more appropriate and tumor-specific neoadjuvant chemotherapy. A small proof-of-concept study [67] developed and applied a PCR assay detecting internal tandem duplications in *BCOR*, a hallmark of clear cell sarcoma of the kidney, to plasma samples. This assay was used to pre-operatively differentiate clear cell sarcoma from nephroblastoma in four patients.

### Brain tumors

Pathological examination is the gold standard for definitive brain tumor diagnosis and subtyping, but this is often challenging. The most recent WHO classification defines molecular parameters in addition to histopathology for diagnosis [42]. Classification of brain tumors by methylation analysis appears to outperform histopathological diagnostics at least for several tumor types [12, 52, 62]. This predicts a major role for ctDNA-based diagnosis of central nervous system tumors, facilitating early management and therapy, especially in cases where tumor localization prevents resection. Cell-free DNA load in blood plasma has only been tested in pediatric patients with medulloblastomas (among brain tumors) to date, where its presence was demonstrated in 40% of patients [7]. Tumor-derived histone H3 gene mutations were detected in blood plasma from pediatric patients with diffuse midline gliomas [31]. Martinez-Ricarte et al*.* were able to classify 17 of 20 patients (including 2 children) with diffuse gliomas by analyzing only 7 genes in cell-free DNA from CSF [44]. Paret et al*.* reported on one pediatric case of neuroepithelial high-grade tumor of the central nervous system showing a BCOR internal duplication, whose detection in plasma cell-free DNA correlated with relapse development [53]. The blood-brain barrier significantly restricts the amount of ctDNA entering the blood [7, 22]. An alternative source of ctDNA for brain tumors is CSF, which has been demonstrated to contain ctDNA to a certain extent in adult patients [58]. Many pediatric patients with brain tumors present with critically elevated intracranial pressure [50], in whom acute neurosurgical intervention is necessary. CSF can be safely obtained for ctDNA analysis during this procedure with no additional risk or burden to the patient. The diagnostic utility of this analysis across the range of both high- and low-grade pediatric brain tumors has not yet been explored. We expect this evidence to emerge within the next years, as techniques for cell-free DNA methylation detection are being further developed [21, 59]. CSF can also be obtained by lumbar puncture; while not minimally invasive, this technique is a relatively safe and often included in routine testing for neurological symptoms in pediatric patients and as a staging tool in brain tumors. When a CNS tumor is suspected, the benefit of a lumbar puncture to obtain CSF for ctDNA analysis might outweigh the risks associated with sampling.

### Retinoblastoma

Although not minimally invasive or easily accessible, the vitreous fluid has been retrospectively examined in 26 patients with retinoblastomas. Tumor-specific copy number alterations and *RB1* mutations detected in the vitreous fluid using shallow whole-genome sequencing strongly correlated with the need for eye enucleation. This testing may become a biomarker to guide the important decision whether to enucleate or salvage the eye in future trials [5, 6]. Blood-based liquid biopsies have not been explored for retinoblastoma.

## Evaluating therapeutic response and clonal evolution

**Fig. 2:**
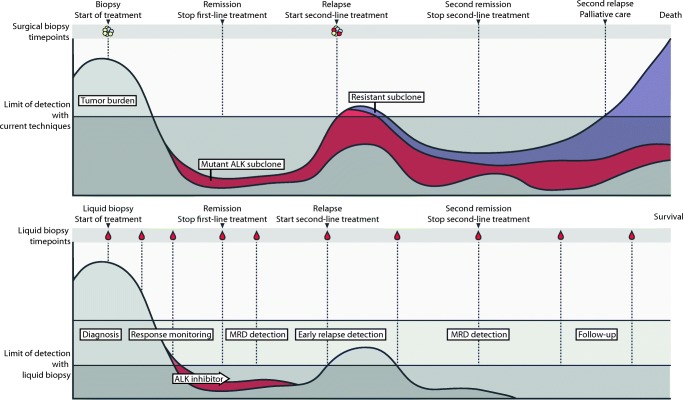
Comparison for a theoretical patient with neuroblastoma: the course under current care protocols (top panel) of a patient with neuroblastoma and the projected course after the routine implementation of liquid biopsy diagnostics (bottom panel). Current treatment protocols evaluate the primary tumor only at diagnosis and relapse. A subclone harboring an *ALK* mutation is able to remain undetected until overt clinical relapse and is able to develop resistant to therapy. The tumor burden can be monitored more continuously using liquid biopsies. The subclone harboring the *ALK* mutation could be detected earlier, and treatment with an ALK inhibitor initiated without first observing a clinical overt relapse. Earlier treatment could possibly prevent a resistant subclone from expanding, thus, potentially improving outcome for the patient

Liquid biopsy–based monitoring of therapy response in pediatric cancer patients has been evaluated in some studies in limited patient numbers, but clear evidence from prospectively validated large studies has not yet been published. Tumor heterogeneity and longitudinal follow-up of single-nucleotide variants and copy number aberrations in circulating cell-free DNA have best been explored to date in a study by Chicard et al. [15]. Using a combination of whole-exome and targeted sequencing of both the primary tumor and plasma samples collected at different time points, the authors demonstrated the subclonal makeup of neuroblastomas and the accumulation of additional genomic alterations during tumor evolution towards therapy-resistant disease. Some alterations were potentially targetable (MAPK pathway) suggesting an application for liquid biopsy diagnostics in therapy decisions and response evaluation [15]. We demonstrate the difference that could be made by liquid biopsy–based monitoring in the clinical course of a theoretical patient with neuroblastoma (Fig. 2).

## Minimal residual disease and early relapse detection

Two major opportunities provided by liquid biopsies are to improve MRD monitoring under primary treatment and the early molecular-based diagnosis of relapse during follow-up. MRD detection is well established for children with acute lymphoblastic leukemia since the beginning of the 2010s, and is now part of routine follow-up in many frontline treatment protocols both to determine optimal treatment intensity and to diagnose relapse prior to the onset of clinical symptoms [11, 32]. Based on the analysis of plasma obtained from 44 pediatric patients with different solid tumors using next-generation sequencing and droplet digital PCR, Kurihara et al. suggest that total ctDNA amount can serve as a marker to evaluate how completely a pediatric tumor is resected following surgery [38]. Whole-genome profiling of primary neuroblastomas was used to generate tumor-specific DNA-based PCR assays for MRD monitoring in blood and bone marrow in eight patients, providing proof-of-concept for this paradigm in solid tumors. The MRD panel was capable of predicting disease relapse or bone marrow progression in four of five patients [69]. Further, different mRNA markers are being explored for MRD detection in blood (*PHOX2B*, *TH*, *DDC*, *DBH*, and *CHRNA3*) and bone marrow (*PHOX2B*, *TH*, *DDC*, *CHRNA3*, and *GAP43*) from neuroblastoma patients [63, 71]. This mRNA-based detection panel can be applied to assess treatment response as well as MRD detection, since high panel transcript levels at diagnosis, after induction therapy, and at the completion of treatment were associated with worse patient outcome [19, 64, 70, 72, 78]. Increasing evidence suggests that treatment follow-up using liquid biopsies is also feasible for patients with Ewing sarcoma. Measurement of the *EWSR1* fusion gene copy numbers in 234 blood samples from 20 patients showed that recurring or increasing levels correlated with relapse [37]. In another study, patient-specific primers for use in droplet digital PCR were established to detect tumor-specific *ESWR-ETS* fusion gene breakpoint fragments in plasma samples from, to date, three patients with Ewing sarcoma. In two of these patients, fusion gene fragments were detected in plasma samples at a time when the disease was radiographically undetectable, altogether suggesting that measuring tumor-specific *EWS-ETS* fusion gene breakpoint fragments in the blood may serve as a reliable personalized biomarker for early relapse detection in patients with Ewing sarcoma [30].

## Limitations and challenges

### Quality issues and standards

The pre-analytical phase, including type and handling of the blood collection tube, storage temperature, time-to-processing and centrifugation speed, all influences the availability and composition of cell-free DNA in the sample [24]. For example, white blood cell lysis further increases their contribution to total cell-free DNA and dilutes the ctDNA fraction. Most studies conducted so far have utilized retrospectively collected plasma and blood samples, preventing investigation of pre-analytical variables that might interfere with the amount of ctDNA or total cell-free DNA. Standardizing pre-analytical variables will be necessary before liquid biopsies can enter routine clinical care. Preservation tubes for cell-free DNA analysis exist and have clearly proven capable of stabilizing cell-free DNA for longer periods [35, 45], but are more expensive and not often on hand in all hospitals. Further testing is necessary to arrive at standards to maintain quality for different liquid biopsy–based assays.

### Physiology of cell-free DNA

Analogous to other pediatric biological variables, it is to be expected that processes regulating the shedding of cell-free DNA into the bloodstream and its metabolism may be different in children and adults, and may even vary between infants, young children, and adolescents. Cell-free DNA levels were shown to be higher in older individuals (19–30 and 67–97 age groups were compared) in the general population, and authors speculated that older people could have difficulty clearing cell-free DNA from the blood [47]. It is conceivable that health, similar to age, has a general impact on plasma cell-free DNA content. Comorbidities can also occur in pediatric cancer patients (e.g., acute and chronic kidney disease, sepsis) that have been shown to elevate total circulating cell-free DNA levels [16]. Data from adult patients show that the ctDNA compartment makes up a significantly higher fraction of circulating cell-free DNA in plasma from patients with high-stage and metastatic disease than low-stage disease [7], indicating liquid biopsies may be more relevant in high-stage and metastatic disease in children with cancer as well. The one study of retrospectively collected plasma samples from patients with multiple pediatric cancer types detected a significantly higher level of cell-free DNA in plasma from patients with neuroblastoma compared with patients with Ewing sarcoma, osteosarcoma, Wilms tumor, and alveolar rhabdomyosarcoma [36]. However, relative ctDNA plasma loads have not yet been thoroughly investigated across pediatric cancer types and stages. Circulating cell-free DNA has been most intensively studied in patients with neuroblastoma to date. Total cell-free DNA levels are approximately 100-fold higher in plasma from patients diagnosed with high-risk neuroblastoma than in healthy adults, indicating that tumor-derived DNA contributes largely to the circulating DNA in high-risk patients. Tumor-derived DNA was estimated to make up between 3 and 99% of circulating cell-free DNA in these patients [15, 36]. While these investigations indicate liquid biopsies may be more beneficial for patients with high-stage or metastatic disease, we should be careful of generalizing from the limited data available at this time. The extent to which patient age, tumor type, disease stage, or other variables impact the uses or usefulness of liquid biopsies will only become clear after their systematic integration in studies accompanying trials.

### Low rates of recurrent genomic alterations common to pediatric cancers

In comparison with adult tumor types, hotspot mutations and recurrent genomic alterations are rare in pediatric cancer [28]. Recurrent genetic alterations or hotspot mutations that are tumor-specific are ideal targets for ctDNA diagnostics or MRD detection [13]. They offer the advantage that highly optimized assays, such as those applying droplet digital PCR, can be developed for their detection. Although altogether rare, some notable exceptions for hotspot mutations and recurrent genomic alterations that impact clinical care exist. This includes *ALK*^*F1174*^ and *ALK*^*R1275*^ mutations and *MYCN* amplifications in primary neuroblastomas [29, 56, 63], the *BRAF*^*V600E*^ mutation in Langerhans cell histiocytosis [3], and *EWSR1* translocations in Ewing sarcoma [20], to name a few.

### Impact on therapy and outcome

Liquid biopsy–based diagnostics, when optimized, can support improved MRD monitoring and early relapse detection and provide information for therapy decisions. The value of monitoring recurrence will remain limited without the availability of adequate relapse treatments, but this limitation also exists for on-going large concerted biology-driven, early phase precision medicine trials for high-risk, relapsed or refractory pediatric cancers (eSMART: NCT02813135; INFORM [77]; Ped-MATCH: NCT03155620; PRISM: NCT03336931; iCat2 from the GAIN Consortium: NCT02520713). All these studies require extensive molecular profiling of relapsed tumor samples before trial entry. Some (early phase) clinical trials for relapsed patients now include ctDNA analysis to follow the evolution of tumor genetics during targeted treatment (eSMART; MAPPYACTS: NCT02613962).

## Discussion and conclusions

Liquid biopsy applications for pediatric oncology are lagging behind their adult counterpart, and studies so far have mostly been retrospective proof-of-concept studies in small cohorts. Nevertheless, these proof-of-concept studies illustrate that the technology yields substantial potential (Table 1). Pediatric tumor types, consisting mainly of highly immature and fast-growing tumor cells, might even be better suited to liquid biopsy–based genomics than many cancers arising in adult patients. Larger and especially prospective clinical trials are needed to fully explore the potential of liquid biopsy–based diagnosis, therapy response monitoring, and residual disease detection. In comparison with adult oncology, where less than 5% of patients are enrolled in a randomized controlled trial [48], the majority of children with cancers are enrolled. The first randomized controlled trials in pediatric oncology that will collect liquid biopsies to explore its potential are currently being initiated (NCT02546453, NCT03496402, NCT03336931). The data generated will hopefully elucidate whether this novel technology is complementary to traditional diagnostic procedures and demonstrate what impact they can have on clinical decision making. We expect to see many novel frontline international treatment protocols make use of liquid biopsy diagnostics in the future. Blood-based liquid biopsies might be the first step, but in due time, could be complemented or replaced by urine [10] or other fluid sources, such as saliva or CSF [46], as has developed for specific applications in the adult population.

**Table 1:** Overview of proof-of-concept studies of liquid biopsies involving pediatric oncology patients

Author	Tumor entity	Number of pediatric patients	Technique	Biomaterial	Method	Clinical application
Chicard [14]	Neuroblastoma	70	OncoScan Array (Affymetrix)	Plasma	CNA profiling	Therapeutic and risk stratification
Van Roy [68]	Neuroblastoma	37	sWGS	Plasma	CNA profiling	Therapeutic and risk stratification
Combaret [17]	Neuroblastoma	114	ddPCR	Plasma	*ALK* hotspot mutation	Diagnostic and therapeutic stratification
Combaret [18]	Neuroblastoma	102	PCR	Plasma, serum	*MYCN* amplification	Diagnostic and therapeutic stratification
Lodrini [41]	Neuroblastoma	10	ddPCR	Plasma	*ALK* and *MYCN* copy number status	Diagnosis and therapeutic stratification, Monitoring disease progression
Chicard [15]	Neuroblastoma	19	WES, ands targeted resequencing	Plasma	SNV and CNA profiling at different timepoints	Identifying treatment-resistant clones, Longitudinal follow-up
Jimenez [33]	Renal tumors	18	WES	Plasma	Somatic SNV and CNA profiling at diagnosis	Improved molecular diagnosis
Ueno-Yokohata [67]	Clear cell sarcoma of the kidney	4	PCR	Plasma	Detection of internal tandem duplication of *BCOR*	Improved molecular diagnosis
Krumbholz [37]	Ewing sarcoma	20	ddPCR	Plasma	*EWSR1* fusion gene detection	Therapy monitoring
Shukla [60]	Ewing sarcoma, Desmoplastic small round cell tumor	7	ddPCR, Targeted resequencing	Plasma	*ESWR1* fusion gene detection	Disease monitoring
Hayashi [30]	Ewing sarcoma	3	ddPCR	Plasma	*EWS-ETS fusion gene detection*	Therapy monitoring
Barris [4]	Osteosarcoma	4	Targeted resequencing	Plasma	Patient-specific alterations in 7 genes	Disease monitoring
Mussolin [49]	Hodgkin and NHL	201	qPCR	Plasma	Presence of cell free DNA	Improved diagnostics
Machado [43]	B-NHL	30	qPCR	Plasma	Total cell-free and EBV virus DNA quantification	Disease detection and treatment, response monitoring
Bruscaggin [9]	Hodgkin lymphoma	44	CAPP-Seq	Plasma	Genotyping of newly diagnosed and refractory HL	Disease monitoring
Primerano [55]	Hodgkin lymphoma	155	qPCR	Plasma	Cell-free DNA quantification	Disease detection and treatment Response monitoring
Berry [5]	Retinoblastoma	6	sWGS, Sanger	Vitreous fluid	CNA profiling, RB1 mutation detection	Surrogate for tumor biopsy after salvage therapy
Berry [6]	Retinoblastoma	26	sWGS	Vitreous fluid	CNA profiling	Therapy response monitoring
Huang [31]	Diffuse midline gliomas	11	Nested PCR, Sanger	CSF	Histone H3 gene mutation in CSF	Alternative or complementary to tissue diagnosis
Martinez-Ricarte [44]	Gliomas	2	Targeted sequencing, ddPCR	CSF	Detection of *IDH1/2, TP53, ATRX, TERT, H3F3A, HIST1H3B* gene mutations in CSF	Facilitating diagnosis of diffuse gliomas
Paret [53]	HGNET-BCOR	1	Patient-specific PCR + Sanger	Plasma	Follow-up of a BCOR internal tandem duplication	Personalized treatment and therapy monitoring
Klega [36]	Osteosarcoma, neuroblastoma, alveolar rhabdomyosarcoma, Wilms tumor	45	sWGS	Plasma	CNA and translocation characterization	Disease detection, Risk stratification, Treatment response monitoring
Weaver [74]	Gliomas	10	Methylation-specific PCR	Plasma	Promoter methylation detection	Disease monitoring

*CSF*, cerebrospinal fluid; *HGNET-BCOR*, high-grade neuroepithelial tumor of the central nervous system with *BCOR* alteration; *PCR*, polymerase chain reaction; *SNV*, single-nucleotide variant; *WES*, whole-exome sequencing; *qPCR*, quantitative PCR; *ddPCR*, droplet digital PCR: *CNA*, copy number alterations; *NHL*, non-Hodgkin lymphoma; *EBV*, Epstein-Barr virus; *sWGS*, shallow whole-genome sequencing; *SV*, structural variant

For specific disease types, liquid biopsy diagnostics may be able to resolve some long-standing issues. The discussion of whether to perform surgery first, or what type of chemotherapy should be applied for renal tumors might become resolved by liquid biopsy–based genomic profiling to improve diagnostics and risk stratification [8]. Identifying a kidney tumor as a Wilms or other tumor entity (e.g., differential diagnosis between Wilms tumor and clear cell sarcoma) from genomic profiling of circulating cell-free DNA at diagnosis would eliminate the need to resort to risky tissue biopsies with the potential to cause tumor rupture. Molecular classification of renal tumors through liquid biopsy approaches might directly impact the clinical course in up to 10% of children who currently succumb to a renal tumor. Additional valuable genomic insights might be available through liquid biopsy–based analysis of diffuse intrinsic pontine gliomas, for which no effective treatment strategies currently exist and biopsy at diagnosis is limited by the risks involved. Improved biological insights could drive development of novel, effective therapies. Liquid biopsies may even have an impact in general pediatrics, for example, in the diagnostic workup of an enlarged lymph node. A well-validated and highly sensitive test to detect lymphoma-associated genetic alterations might reduce the number of lymph node biopsies necessary to diagnose lymphadenitis as opposed to lymphoma. We expect the clinical impact of liquid biopsy diagnostics to become clear in the field of pediatric oncology in the coming decade. Well-organized prospective multicenter trials will be necessary to further delineate the potential applications and clinical utility of this novel technology.

## Abbreviations

CSF: Cerebrospinal fluid
ctDNA: Circulating or cell-free tumor DNA
MRD: Minimal residual disease
PCR: Polymerase chain reaction)
